# The ratios of central venous to arterial carbon dioxide content and tension to arteriovenous oxygen content are not associated with overall anaerobic metabolism in postoperative cardiac surgery patients

**DOI:** 10.1371/journal.pone.0205950

**Published:** 2018-10-26

**Authors:** Osama Abou-Arab, Rayan Braik, Pierre Huette, Belaid Bouhemad, Emmanuel Lorne, Pierre-Grégoire Guinot

**Affiliations:** 1 Anaesthesiology and Critical Care Department, Amiens University Hospital, Rond-point Fernand Leger, Amiens, France; 2 Anaesthesiology and Critical Care Department, Dijon University Hospital, 2 Bd Maréchal de Lattre de Tassigny, Dijon, France; Boston University, UNITED STATES

## Abstract

**Background:**

The aim of the present study was to evaluate the ability of the ratios of central venous to arterial carbon dioxide content and tension to arteriovenous oxygen content to predict an increase in oxygen consumption (VO_2_) upon fluid challenge (FC).

**Methods and results:**

110 patients admitted to cardiothoracic ICU and in whom the physician had decided to perform an FC (with 500 ml of Ringer's lactate solution) were included. The arterial pressure, cardiac index (Ci), and arterial and venous blood gas levels were measured before and after FC. VO_2_ and CO_2_-O_2_ derived variables were calculated. VO_2_ responders were defined as patients showing more than a 15% increase in VO_2_. Of the 92 FC responders, 43 (46%) were VO_2_ responders. At baseline, pCO_2_ gap, C(a-v)O_2_ were lower in VO_2_ responders than in VO_2_ non-responders, and central venous oxygen saturation (ScvO_2_) was higher in VO_2_ responders. FC was associated with an increase in MAP, SV, and CI in both groups. With regard to ScvO_2,_ FC was associated with an increase in VO_2_ non-responders and a decrease in VO_2_ responders. FC was associated with a decrease in pvCO_2_ and pCO_2_ gap in VO_2_ non-responders only. The pCO_2_ gap/C(a-v)O_2_ ratio and C(a-v)CO_2_ content /C(a-v)O_2_ content ratio did not change with FC. The CO_2_ gap content/C(a-v)O_2_ content ratio and the C(a-v)CO_2_ content /C(a-v)O_2_ content ratio did not predict fluid-induced VO_2_ changes (area under the curve (AUC) [95% confidence interval (CI)] = 0.52 [0.39‒0.64] and 0.53 [0.4–0.65], respectively; p = 0.757 and 0.71, respectively). ScvO_2_ predicted an increase of more than 15% in the VO_2_ (AUC [95%CI] = 0.67 [0.55‒0.78]; p<0.0001).

**Conclusions:**

Our results showed that the ratios of central venous to arterial carbon dioxide content and tension to arteriovenous oxygen content were not predictive of VO_2_ changes following fluid challenge in postoperative cardiac surgery patients.

## Introduction

Fluid challenge (FC) is the most frequently performed bedside haemodynamic intervention in perioperative care. This procedure is usually used to increase cardiac output (CO) so that oxygen delivery (DO_2_) matches oxygen consumption (VO_2_) [1, 2]. After FC, VO_2_ can either increase (if there is an oxygen debt) or remain unchanged [2]. In recent years, several studies have focused on parameters that are able to accurately track VO_2_/DO_2_ dependency [3–7]. Although the blood lactate concentration was initially described as a surrogate marker of VO_2_/DO_2_ dependency, an elevated lactate value may not necessarily reflect anaerobic metabolism [8]. Although ScvO_2_ might be indicative of DO_2_, its significance may be diminished during distributive shock with alteration of the oxygen extraction ratio (O_2_ER)—even after cardiac surgery [5, 9, 10]. It was recently suggested that the veno-arterial carbon dioxide tension gradient (pCO_2_ gap) and the pCO_2_ gap/C(a-v)O_2_ ratio are more sensitive indices of anaerobic metabolism and the VO_2_ increase upon FC [5, 11–14]. These parameters were developed and validated in ICU patients with sepsis, in whom they accurately predict an increase in VO_2_ with FC.

In clinical practice, the difficulty is to identify hemodynamic and/or oxygenation parameters that are clinically relevant to become endpoints for titration of interventions. Increasing DO_2_ is an accepted goal for optimization following cardiac surgery [15, 16] which is considered as a major surgery associated with high incidence of postoperative complications. Thus, predicting VO_2_ responsiveness can identify the patients for which DO_2_ increase is most beneficial [15, 16]. To date, these parameters have not been extensively studied in non-septic or post-operative patients. A few studies of postoperative cardiac surgery patients have shown that in contrary to the situation in patients with sepsis, pCO_2_ gap is poorly correlated with perfusion variables [17, 18].

The present study aims at investigating the ability of the pCO_2_ gap/C(a-v)O_2_ ratio and the C(a-v)CO_2_ content/C(a-v)O_2_ content ratio to predict a VO_2_ increase upon FC in postoperative cardiac surgery patients.

## Material and methods

### Ethics

The study was approved by the independent ethics committee at Amiens University Hospital (Amiens, France). Because the protocol study is considered as observational and part of routine clinical practice, the French law did not require written consent. According to ethics committee, all patients received written information on the study. Oral consent was obtained from patient or subject’s next of kin. The capacity to consent was checked by excluding confusion in awake patient who were not sedated. Confusion was assessed by clinical examination based on confusion assessment method for the intensive care unit. In case of confusion, the consent was obtained from subject’s of kin. The consent was noted on study observation book. The present manuscript was drafted in compliance with the STROBE checklist for cohort studies [19].

### Patients

This observational study was performed in the cardiothoracic ICU at Amiens University Hospital (Amiens, France) between 2014 and 2017. Some of the patients were previously included in a study that evaluate association between end tidal carbon dioxide pressure and oxygen extraction [7]. The main inclusion criteria were as follows: age 18 or over, controlled positive ventilation, and a clinical decision to perform FC for volume expansion. The indications for FC were arterial hypotension (a systolic arterial pressure (SAP) below 90 mmHg and/or a mean arterial pressure (MAP) below 65 mmHg), a stroke volume (SV) variation of more than 10% during a passive leg raising manoeuver and/or clinical signs of hypoperfusion (skin mottling, and a capillary refill time of more than 3 sec). The non-inclusion criteria were permanent arrhythmia, heart conduction block, a pacemaker, poor echogenicity, aortic regurgitation, spontaneous ventilation, ongoing haemorrhage, and right heart dysfunction.

### Haemodynamic parameters

Transthoracic echocardiography (with the CX50 ultrasound system and an S5-1 Sector Array Transducer, Philips Medical System, Suresnes, France) was performed by a physician who was blinded to the study outcomes. The left ventricular ejection fraction was measured using Simpson’s biplane method with a four-chamber view. The aortic surface area (SAo, in cm^2^) was calculated as π×(diameter of the left ventricular outflow tract)^2^/4. The aortic velocity-time integral (VTIAo), was measured with pulsed Doppler at the LVOT on a five-chamber view. The SV (mL) was calculated as VTIAo×SAo. Cardiac output (CO) was calculated as SV×heart rate (HR) (ml min^-1^) and was expressed as an indexed CI, i.e. CO/body surface area (ml min^-1^ m^2^). Mean echocardiographic parameters were calculated from five measurements (regardless of the respiratory cycle) and analysed off lines.

### Oxygenation parameters

We recorded the ventilator settings (tidal volume, plateau pressure and end-expiratory pressure) at baseline. All blood gas parameters were measured with arterial and central venous catheters. Arterial and venous blood gas levels, the blood lactate level, the blood haemoglobin (Hb) concentration and oxyhaemoglobin saturation were measured using an automated analyser (ABL800 FLEX, Radiometer, Bronshoj, Denmark). Arterial oxygen content (CaO_2_) and venous oxygen content (CvO_2_) were calculated as follows: CaO_2_ = 1.34 x Hb x SaO_2_ + 0.003 x PaO_2_; CvO_2_ = 1.34 x Hb x ScvO_2_ + 0.003 x PvO_2_, where Hb is the haemoglobin concentration (g.dl^-1^), PaO_2_ is the arterial oxygen pressure (mmHg), SaO_2_ is the arterial oxygen saturation (%), PvO_2_ is the venous oxygen pressure (mmHg), ScvO_2_ is the central venous oxygen saturation (in%), and 0.003 is the solubility coefficient of oxygen [14]. pCO_2_ gap was calculated as follows: pCO_2_ gap = PcvCO_2_ –PaCO_2_ (mmHg). C(a-v)O_2_ was calculated as CaO_2_ minus CvO_2_ (ml) [14]. DO_2_ and VO_2_ were calculated from arterial and central venous blood gas measurements as follows: DO_2_ (ml min^-1^ m^-2^) = (CaO_2_ x 10 x CO)/body surface area; VO_2_ (ml min^-1^ m^-2^) = the arteriovenous difference in oxygen content (C(a-v)O_2_ x CO x 10)/body surface area. Arterial and venous CO_2_ contents (CaCO_2_, CvCO_2_) were calculated according to the Douglas formula [14, 20]. The C(a-v)CO_2_ content was calculated as CvCO_2_ minus CaCO_2_ (ml).

### Protocol

During the study period, the patients were mechanically ventilated in volume-controlled mode, with a tidal volume set to 7–9 ml kg^-1^ ideal body weight, and a positive end-expiratory pressure (PEEP) of 5–8 cmH_2_O. The patients were sedated with propofol, with a target Ramsay score >5. The ventilator settings (oxygen inspired fraction, tidal volume, respiratory rate, and end positive pressure) were not modified during the study period.

The following clinical parameters were recorded: age, gender, weight, ventilation parameters, and primary diagnosis. After an equilibration period, HR, SAP, MAP, diastolic arterial pressure, central venous pressure (CVP), SV, CO, and arterial/venous blood gas levels were measured at baseline. In the present study, FC always consisted of a 10-minute infusion of 500 ml of Ringer's lactate solution. Immediately after FC, a second set of measurements was made.

### Statistical analysis

The variables' distribution was assessed using a Shapiro-Wilk test. Data were expressed as the number, proportion (in percent), mean ± standard deviation (SD) or the median [interquartile range (IQR)], as appropriate. Patients were classified as fluid responders or non-responders as a function of the effect of FC on the SV. An FC response was defined as an increase of more than 15% in the SV after FC [21]. Patients were classified as VO_2_ responders or non-responders as a function of the effect of FC on VO_2_. A VO_2_ response was defined as an increase of more than 15% in the VO_2_ after FC [7]. The non-parametric Wilcoxon rank sum test, Student’s paired t test, Student’s t test, and the Mann-Whitney test were used to assess statistical significance, as appropriate. Linear correlations were tested using Pearson's or Spearman's rank method. A receiver-operating characteristic curve was used to establish the ability of ScvO_2_, pCO_2_ gap/C(a-v)O_2_ ratio or the C(a-v)CO_2_ content/C(a-v)O_2_ content ratio to predict an increase of more than 15% in VO_2_ [7, 14]. Assuming that 60% of patients would be fluid responders and that 20 to 30% of fluid responders would be VO_2_ responders, we calculated that a sample of 105 patients was sufficient to demonstrate that the pCO_2_ gap/C(a-v)O_2_ ratio predict an increase in VO_2_ upon FC with an area under the curve (AUC) greater than 0.80, a power of 80%, and an alpha risk of 0.05. Taking the exclusion criteria and incomplete data in account, the sample size was set to 115 participants. The threshold for statistical significance was set to *p*<0.05. SPSS software (version 24, IBM, New York, NY, USA) was used for all statistical analyses.

## Results

### Patients

**Fig 1:**
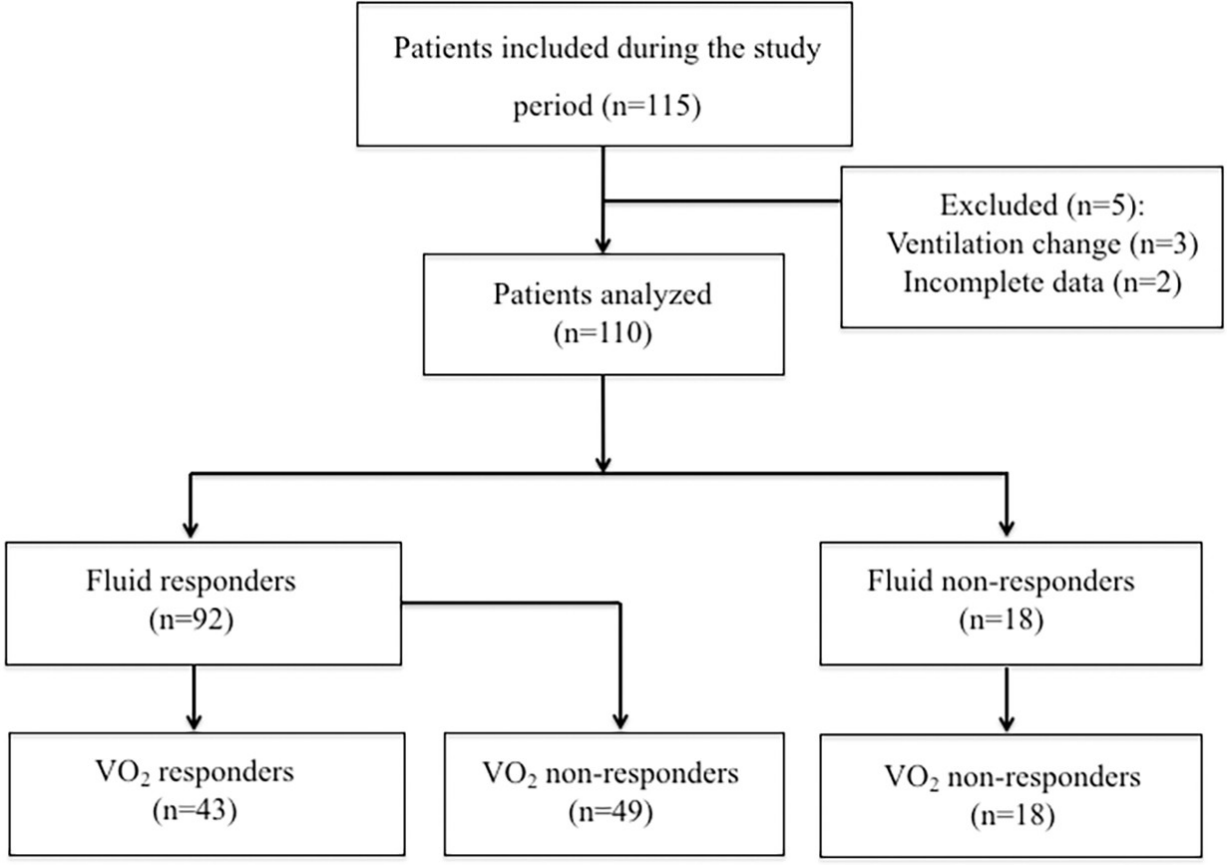
Flow chart diagram of the study.

All patients had undergone cardiovascular surgery with cardiopulmonary bypass Table 1, Fig 1. Of the 115 included patients, five were excluded (Fig 1), and so the final analysis covered 110 patients. Of these, 92 (84%) were classified as FC responders, and 43 (47%) were classified as VO_2_ responders.

**Table 1:** Characteristics of the study participants on inclusion.

Variables	Overall population (n = 110)
Age (mean (SD), years)	69 (11)
Gender (F/M)	32 /78
Surgery, n (%)	
Valvular	55 (50)
CABG	30 (27)
Combined surgery	15 (14)
Other	6 (9)
SAPS 2	40 (13)
Respiratory parameters	
Tidal volume (ml kg^-1^ of predicted body weight, mean (SD),	7.8 (0.6)
Total PEEP (cmH_2_O, mean (SD))	6 (1)
Number of patients treated with norepinephrine (n, %)	25 (25)
Median dose (gamma Kg^-1^ min^-1^)	0.7 (0.5 to 1.4)
Number of patients treated with dobutamine (n, %)	4 (5)
Median dose (gamma Kg^-1^ min^-1^)	5 (5 to 7)
LVEF (%, mean (SD))	49 (11)

Values are expressed as the mean ± SD or the number (%). CABG: coronary artery bypass graft.

### Effect of FC on haemodynamic and oxygenation parameters in the population as a whole

FC was associated with increases in MAP, CVP, SV, CO, DO_2_, and VO_2_, and decreases in HR, and pCO_2_ gap Table 2. At baseline, the arterial lactate concentration was not correlated with ScvO_2_ (r = -0.044, p = 0.650), pCO_2_ gap/C(a-v)O_2_ ratio (r = 0.052, p = 0.587), or C(a-v)CO_2_ content /C(a-v)O_2_ content ratio (r = 0.019, p = 0.841).

**Table 2:** Comparison of haemodynamic parameters according to response of VO_2_.

Hemodynamic variables	VO_2_ responders (n = 43)	VO_2_ non responders (n = 49)	*p value*
Respiratory minute ventilation (l min^-1^)	**8.2 (1.3)**	**8 (1)**	***0*.*290***
Body temperature (°C)	**36.3 (1.7)**	**36.6 (0.4)**	***0*.*273***
Capillary refill time (sec)			
Pre-FC	**3.6 (1.5)**	**3.6 (1.3)**	***0*.*908***
Post-FC	**3.2 (1.2)** ^a^	**2.9 (1.4)** ^a^	***0*.*289***
Haemoglobin (g dl^-1^)			
Pre-FC	**11.4 (1.6)**	**11.2 (1.4)**	***0*.*518***
Post-FC	**11.2 (1.7)** ^a^	**10.8 (1.4)** ^a^	***0*.*210***
HR (bpm)			
Pre-FC	**82 (22)**	**85 (19)**	***0*.*574***
Post-FC	**78 (21)** ^a^	**81 (16)** ^a^	***0*.*404***
MAP (mmHg)			
Pre-FC	**74 (14)**	**70 (12)**	***0*.*140***
Post-FC	**84 (16)** ^a^	**82 (12)** ^a^	***0*.*472***
SV (ml)			
Pre-FC	**44 (15)**	**42 (15)**	***0*.*652***
Post-FC	**60 (18)** ^a^	**55 (21)** ^a^	***0*.*263***
CI (ml min^-1^ m^-2^)			
Pre-FC	**1.7 (0.6)**	**1.8 (0.7)**	***0*.*524***
Post-FC	**2.3 (0.7)** ^a^	**2.2 (0.9)** ^a^	***0*.*921***
DO_2_ (ml min^-1^ m^-2^)			
Pre-FC	**269 (103)**	**274 (95)**	***0*.*811***
Post-FC	**339 (124)** ^a^	**319 (119)** ^a^	***0*.*428***
VO_2_ (ml min^-1^ m^-2^)			
Pre-FC	**75 (34)**	**100 (39)**	***0*.*002***
Post-FC	**115 (37)** ^a^	**93 (31)**	***0*.*007***

Values are expressed as the mean (SD) or the median [interquartile range]. **CI**, indexed cardiac output; ***DO***_***2***_, oxygen delivery; **FC**, fluid challenge; **HR**, heart rate; **MAP**, mean arterial pressure; **SV**, stroke volume; ***VO***_***2***_, oxygen consumption

^**a**^: *p*<0.05 within groups (pre-/post-FC).

### Differences between VO_2_ responders and VO_2_ non-responders among fluid responders

Of the 92 FC responders, 43 (46%) were VO_2_ responders (Fig 1). All VO_2_ responders were FC responders Table 2. FC increased MAP, SV, and CI in the two groups Table 2.

At baseline, pCO_2_ gap and C(a-v)O_2_ were lower in VO_2_ responders than in VO_2_ non-responders, and ScvO_2_ was higher Table 3. The arterial lactate concentration did not differ when comparing the two groups, and did not change upon FC. Furthermore, FC increased ScvO_2_ in VO_2_ non-responders and decreased ScvO_2_ in VO_2_ responders. FC decreased pvCO_2_ and pCO_2_ gap in VO_2_ non-responders only Table 3. The pCO_2_ gap/C(a-v)O_2_ ratio and the C(a-v)CO_2_ content/C(a-v)O_2_ content ratio did not change upon FC.

**Table 3:** Comparison of perfusion parameters according to response of VO_2_.

Variables	VO_2_ responders (n = 43)	VO_2_ non responders (n = 49)	*p value*
**Arterial pH**			
**Pre-FC**	**7.35 (0.07)**	**7.38 (0.2)**	***0*.*447***
**Post-FC**	**7.38 (0.05)** ^a^	**7.39 (0.2)**	***0*.*667***
**Venous pH**			
**Pre-FC**	**7.32 (0.05)**	**7.33 (0.2)**	***0*.*751***
**Post-FC**	**7.32 (0.06)**	**7.33 (0.2)**	***0*.*728***
**Oxygen arterial saturation (%)**			
**Pre-FC**	**97.6 (1.2)**	**97.7 (1.7)**	***0*.*679***
**Post-FC**	**97.4 (1.7)**	**97.6 (1.4)**	***0*.*628***
**ScvO**_**2**_ **(%)**			
**Pre-FC**	**67.7 (12)**	**60.8 (10)**	***0*.*003***
**Post-FC**	**62.8 (9)** ^a^	**68.4 (10)** ^a^	***0*.*005***
**PaCO**_**2**_ **(mmHg)**			
**Pre-FC**	**38.4 (5)**	**36.4 (5)**	***0*.*068***
**Post-FC**	**37.3 (4)**	**36.7 (5)**	***0*.*510***
**PvCO**_**2**_ **(mmHg)**			
**Pre-FC**	**46.7 (6.1)**	**46.6 (5.4)**	***0*.*942***
**Post-FC**	**46.5 (5.4)**	**44.7 (5.3)** ^a^	***0*.*104***
**pCO**_**2**_ **gap (mmHg)**			
**Pre-FC**	**8.3 (3.7)**	**10 (3.3)**	***0*.*020***
**Post-FC**	**9.2 (3.8)**	**8 (3.6)** ^a^	***0*.*143***
**CaO**_**2**_ **(ml)**			
**Pre-FC**	**15.4 (2.2)**	**15.1 (2)**	***0*.*555***
**Post-FC**	**15 (2.2)** ^a^	**14.4 (1.9)** ^a^	***0*.*171***
**CvO**_**2**_ **(ml)**			
**Pre-FC**	**10.8 (2.7)**	**9.5 (2.1)**	***0*.*009***
**Post-FC**	**9.7 (2.2)** ^a^	**10.2 (2.2)** ^a^	***0*.*285***
**C(a-v)O**_**2**_ **(ml)**			
**Pre-FC**	**4.5 (1.8)**	**5.6 (1.6)**	***0*.*003***
**Post-FC**	**5.3 (1.2)** ^a^	**4.2 (1.9)** ^a^	***0*.*002***
**CaCO**_**2**_ **(ml)**			
**Pre-FC**	**51.2 (7)**	**48.3 (7.9)**	***0*.*034***
**Post-FC**	**52.1 (5.9)**	**49.8 (5.1)** ^a^	***0*.*052***
**CvCO**_**2**_ **(ml)**			
**Pre-FC**	**57.3 (5.8)**	**55.6 (5.4)**	***0*.*052***
**Post-FC**	**56.9 (6.3)**	**53.2 (5.9)** ^a^	***0*.*004***
**C(a-v)CO**_**2**_ **content (ml)**			
**Pre-FC**	**5.8 (2.9–7.4)**	**6.8 (4.5–7.4)**	***0*.*239***
**Post-FC**	**5.3 (3.5–7.3)**	**2.9 (1.6–6.1)** ^a^	***0*.*023***
**pCO**_**2**_ **gap/C(a-v)O**_**2**_ **(mmHg ml**^**-1**^**)**			
**Pre-FC**	**1.93 (1.36–2.29)**	**1.89 (1.42–2.)**	***0*.*710***
**Post-FC**	**1.82 (1.39–2.21)**	**1.86 (1.36–2.29)** ^a^	***0*.*863***
**C(a-v)CO**_**2**_ **content /C(a-v)O**_**2**_ **content ratio**			
**Pre-FC**	**0.98 (0.43–2.06)**	**1.1 (0.86–1.85)**	***0*.*625***
**Post-FC**	**0.96 (0.59–1.39)**	**0.81 (0.46–1.15)** ^a^	***0*.*109***
**Arterial lactates (mmol l**^**-1**^**)**			
**Pre-FC**	**1.8 (0.9)**	**1.9 (0.7)**	***0*.*590***
**Post-FC**	**1.8 (0.9)**	**2 (0.8)**	***0*.*251***

Values are expressed as the mean (SD) or the median [interquartile range]. **FC**, fluid challenge; ***VO***_***2***_, oxygen consumption

^**a**^: *p*<0.05 within groups (pre-/post-FC).

The FC-induced changes in the C(a-v)CO_2_ content/C(a-v)O_2_ content ratio and the pCO_2_ gap/C(a-v)O_2_ ratio were associated (r = 0.499, p<0.0001), but neither was correlated with changes in VO_2_ (r = -0.092, p = 0.337 and r = -0.05, p = 0.957) or arterial lactates (r = 0.129, p = 0.18 and r = -0.10, p = 0.916). The FC-induced changes in VO_2_ and ScvO_2_ were associated (r = 0.61, p = 0.0001).

### Ability of overall perfusion parameters to predict an increase in VO_2_

With an AUC [95% confidence interval (CI)] of 0.52 [0.39‒0.64] and 0.53 [0.4–0.65], respectively; p = 0.757 and 0.71, respectively, the C(a-v)CO_2_ content /C(a-v)O_2_ content ratio and the pCO_2_ gap/C(a-v)O_2_ ratio did not predict FC-associated changes in VO_2_. Baseline ScvO_2_ was poorly predictive of an increase of more than 15% in the VO_2_, with an AUC [95%CI] of 0.67 [0.55‒0.78] (p<0.0001).

## Discussion

Our study produced several relevant results. The pCO_2_ gap/C(a-v)O_2_ ratio and the C(a-v)CO_2_ content /C(a-v)O_2_ content ratio did not predict increase in VO_2_ in postoperative cardiac surgery patients. ScvO_2_ was poorly predictive of an FC-associated increase in VO_2_. The arterial lactate level was not associated with VO_2_ changes. These results suggest that physician should take in account the population studied before analysing oxygen derivate parameters and predicting VO_2_ dependency.

The pCO_2_ gap/C(a-v)O_2_ ratio and the C(a-v)CO_2_ content/C(a-v)O_2_ content ratio are known to be associated with anaerobic metabolism, lactate clearance, and mortality in ICU patients with sepsis [11, 12, 14]. The present study is the first to have specifically focused on postoperative patients. Our present results did not suggest that the above-mentioned ratios are of value in non-septic patients. There are several possible explanations for our findings. Most of these are probably related to the difference between the various study populations (i.e. sepsis vs cardiac surgery), which may alter the significance of and relationships between systemic parameters related to oxygen and carbon dioxide [9, 22].

In the present study, the relationship between FC and changes in arterial and venous carbon dioxide content/tension differed to that observed in patients with sepsis [6, 12, 14]. Baseline pCO_2_ gap was higher after cardiac surgery in VO_2_ non-responders, and decreased only in VO_2_ non-responders. In the context of sepsis, pCO_2_ gap is higher in VO_2_ responder patients, and decreases only in VO_2_ responder patients. We did not demonstrate differences in FC-induced changes in O_2_-derived parameters, relative to those observed in patients with sepsis. C(a-v)O_2_ decreased in VO_2_ non-responders (due to an increase in CvO_2_) and increased in VO_2_ responders (due to a decrease in CvO_2_). The physiological relationships that allow the pCO_2_ gap/C(a-v)O_2_ ratio and the C(a-v)CO_2_ content/C(a-v)O_2_ content ratio to be used as indicators of anaerobic metabolism are probably altered by the inability of pCO_2_ gap to adequately reflect tissue CO_2_ production and elimination [17]. Our group has already studied pCO_2_ gap as a prognostic factor for the postoperative course in cardiac surgery [17]. Even though pCO_2_ gap was poorly correlated with tissue perfusion parameters, we did not demonstrate an association between pCO_2_ gap and outcomes.

The divergence between sepsis and post-operative situations might be due to several factors. The extent of microcirculation alterations caused by sepsis or surgery/cardiopulmonary bypass may differ [23, 24]. It has been demonstrated that sepsis is systematically associated with the disruption of microcirculatory regulation, i.e. a decrease in the functional capillary index, absent/intermittent capillary flow, increased heterogeneity in the perfusion index, arteriovenous shunting, and cellular hypoxia [25]. Cardiac surgery with cardiopulmonary bypass is associated with a wide range of microcirculatory alterations, including a decrease in microvascular perfusion, increased heterogeneity in the perfusion index and red blood cell velocity, and arteriovenous shunting [23, 26]. These changes are associated with alterations in the arteriovenous oxygen difference, systemic oxygen consumption, and CO_2_ and O_2_ diffusion [27]. Moreover, cardiac surgery microcirculatory alterations may be induced by (amongst other factors) cardiopulmonary bypass haemodilution and temperature changes during the operative period. Haemodilution was demonstrated to alter the relationship between CO_2_ pressures and CO_2_ contents, which do not alter pCO_2_ gap in the same way as haemorrhage [28]. It was also demonstrated that anaesthetic agents alter regional critical DO_2_ and microcirculation by changing the peripheral vascular resistance [29]. When considering the above-mentioned arguments and data as a whole, the pCO_2_ gap/C(a-v)O_2_ ratio and the C(a-v)CO_2_ content/C(a-v) O_2_ content ratio do not reflect complex, inconsistent alterations in regional VO_2_, DO_2_ and the latter’s interrelationships after cardiac surgery.

Our results confirmed those report by Fischer et al., who demonstrated that only ScvO_2_ was associated with VO_2_ dependency in postoperative patients after maximization of the SV by FC [30]. Nevertheless, ScvO_2_ remains poorly predictive of VO_2_ changes [10]. Our results and those of Fischer et al. confirm previous demonstrations of ScvO_2_’s poor ability to track VO_2_ changes [10]. Likewise, arterial lactate was not associated with VO_2_ changes in Fischer et al.’s study and in the present study. Arterial lactate is known to be a complex variable that may be not always be associated with tissue hypoxia/hypoperfusion and anaerobic metabolism [8]. At present, no clinical parameter has demonstrated its superiority to predict VO_2_ dependency. Only goal directed hemodynamic optimisation protocols have demonstrated a decrease of post-operative complications due to a maximisation of DO_2_. Further research is needed to identify and describe new indicators of VO_2_ dependency in non-septic patients. In this way, ventriculo-arterial coupling and mitochondrial PO_2_ may be of interest [31, 32].

The present studies had several limitations. The fact that pCO_2_ gap was measured in central venous blood (rather than mixed venous blood) might have underestimated CO_2_ exchange from splanchnic territories. However, other studies have used central venous blood to calculate VO_2_- and CO_2_-derived parameters [14]. The observed changes in O_2_- and CO_2_-derived parameter were small and reproducible [33]. We assessed VO_2_ using the Fick method, which may not be reliable in ICU patients. Nevertheless, previous studies have used the Fick method to calculate VO_2_ [6, 14]. The latter results were similar to those previously demonstrated to be predictive of VO_2_ changes. Lastly, we performed a single-centre study; however, our results are in line with those reported in Fischer et al.’s study [28].

## Conclusions

Our present results did not demonstrate the ability of the pCO_2_ gap/C(a-v)O_2_ ratio and C(a-v)CO_2_ content/C(a-v)O_2_ content ratio to predict VO_2_ dependency in postoperative cardiac surgery patients. The present finding demonstrated that the population studied should be consider at bedside when assessing VO_2_ dependency with oxygen derivate parameters. The effect of cardiac surgery and/or cardiopulmonary bypass on the relationship between CO_2_ content and CO_2_ partial pressure may explain in part this finding.
